# A scoping review on the significance of programmed death-ligand 1-inhibiting microRNAs in non-small cell lung treatment: A single-cell RNA sequencing-based study

**DOI:** 10.3389/fmed.2022.1027758

**Published:** 2022-10-31

**Authors:** Mahdi Abdoli Shadbad, Farid Ghorbaninezhad, Hamidreza Hassanian, Noora Karim Ahangar, Negar Hosseinkhani, Afshin Derakhshani, Najibeh Shekari, Oronzo Brunetti, Nicola Silvestris, Behzad Baradaran

**Affiliations:** ^1^Student Research Committee, Tabriz University of Medical Sciences, Tabriz, Iran; ^2^Immunology Research Center, Tabriz University of Medical Sciences, Tabriz, Iran; ^3^Laboratory of Experimental Pharmacology, Istituto Di Ricovero e Cura a Carattere Scientifico Istituto Tumori Giovanni Paolo II, Bari, Italy; ^4^Medical Oncology Unit, IRCCS Istituto Tumori “Giovanni Paolo II” of Bari, Bari, Italy; ^5^Medical Oncology Unit, Department of Human Pathology “G. Barresi, University of Messina, Messina, Italy; ^6^Department of Immunology, Faculty of Medicine, Tabriz University of Medical Sciences, Tabriz, Iran

**Keywords:** non-small-cell lung carcinoma, PD-L1, microRNAs, circular RNA, single-cell RNA sequencing

## Abstract

**Background:**

The programmed death-ligand 1 (PD-L1)/PD-1 axis is one of the well-established inhibitory axes in regulating immune responses. Besides the significance of tumor-intrinsic PD-L1 expression in immune evasion, its oncogenic role has been implicated in various malignancies, like non-small cell lung cancer (NSCLC). As small non-coding RNAs, microRNAs (miRs) have pivotal roles in cancer biology. The current study aimed to systematically review the current knowledge about the significance of PD-L1-inhibiting miRs in NSCLC inhibition and their underlying mechanisms.

**Materials and methods:**

We conducted the current scoping review based on the PRISMA-ScR statement. We systematically searched Embase, Scopus, Web of Science, PubMed, Ovid, EBSCO, ProQuest, Cochrane Library, African Index Medicus, and Pascal-Francis up to 4 April 2021. We also performed *in silico* tumor bulk RNA sequencing and single-cell RNA sequencing to further the current knowledge of the non-coding RNA-mediated tumor-intrinsic PD-L1 regulation and the PD-L1/PD-1 axis in NSCLC.

**Results:**

The ectopic expression of hsa-miR-194-5p, hsa-miR-326, hsa-miR-526b-3p, hsa-miR-34a-5p, hsa-miR-34c-5p, hsa-miR-138-5p, hsa-miR-377-3p, hsa-let-7c-5p, hsa-miR-200a-3p, hsa-miR-200b-3p, hsa-miR-200c-3p, and hsa-miR-197-3p, as PD-L1-inhibiting miR, inhibits NSCLC development. These PD-L1-inhibiting miRs can substantially regulate the cell cycle, migration, clonogenicity, invasion, apoptosis, tumor chemosensitivity, and host anti-tumoral immune responses. Based on single-cell RNA sequencing results, PD-L1 inhibition might liberate the tumor-infiltrated CD8^+^ T-cells and dendritic cells (DCs)-mediated anti-tumoral immune responses *via* disrupting the PD-L1/PD-1 axis.

**Conclusion:**

Given the promising preclinical results of these PD-L1-inhibiting miRs in inhibiting NSCLC development, their ectopic expression might improve NSCLC patients’ prognosis; however, further studies are needed to translate this approach into clinical practice.

## Introduction

Lung cancer is projected to be the leading cause of cancer death in the United States; it has been estimated that lung cancer will be responsible for 350 deaths each day in 2022. Also, lung cancer will be the second most commonly affected cancer in both males and females in 2022. Besides, the 5-year relative survival of lung cancer patients is 22% in all races and stages (1). It has been reported that approximately 85% of lung cancer cases are grouped into NSCLCs; lung adenocarcinoma (LUAD) and lung squamous cell carcinoma (LUSC) are the most common subtypes of NSCLC (2). Considering the prevalence, associated burden, and poor survival of affected patients, there is a pressing need to identify novel therapeutic approaches to improve the prognosis of the patients.

Cancer immunotherapy is a novel therapeutic approach that leverages the host immune system to reject tumors (3). The immunosuppressive tumor microenvironment shields malignant cells from anti-tumoral immune responses and reprograms the residing immune cells in a way that they contribute to tumor growth and migration (4). Growing evidence has highlighted the significance of pathological expression of inhibitory immune checkpoint molecules in suppressing anti-tumoral immune responses (5). The PD-L1/PD-1 is one of the well-studied inhibitory immune checkpoint axes that can regulate immune responses. It has been reported that the increased expression of PD-L1 is associated with decreased overall survival and recurrence-free survival of NSCLC patients (6). Following the significant role of the PD-L1/PD-1 axis in facilitating tumor growth, various monoclonal antibodies targeting this axis have been developed, investigated, and approved for NSCLC patients (7). Besides the immunosuppressive role of tumor-intrinsic PD-L1, it is implicated in stimulating oncogenic signaling pathways. For instance, increased expression of PD-L1 in NSCLC cells increases tumor proliferation and colony numbers; however, PD-L1 depletion reserves these pro-tumoral effects in NSCLC cells (8). In cisplatin-treated NSCLC cells, PD-L1 knockdown decreases cell proliferation, migration, and invasion of NSCLC cells (6). It has been shown that the mitogen-activated protein kinase (MAPK) pathway stimulates PD-L1 expression in NSCLC cells, and inhibiting the MAPK pathway decreases PD-L1 protein and mRNA expression (9). Besides, the epidermal growth factor receptor (EGFR)-mediated pathway, as an oncogenic signaling pathway, upregulates PD-L1 expression in NSCLC cells (10). In colorectal cancer cells, PD-L1 knockdown has decreased the protein and mRNA expression of phosphatidylinositol-3-kinase and protein kinase B (PI3K) and protein kinase B (Akt), which are the essential factors of the PI3K/Akt oncogenic signaling pathway (11). Therefore, PD-L1 is considerably involved in oncogenesis and tumor development. Despite the promising results of administrating monoclonal antibodies against the PD-L1/PD-1 axis for NSCLC patients, these antibodies primarily target the PD-L1/PD-1 expression on various cells. Indeed, they are not designed to inhibit the mRNA or protein expression of oncogenic signaling pathway elements in malignant cells (12).

miRs are small non-coding RNAs that have considerable roles in the post-transcriptional regulation of gene expression (13). These non-coding RNAs substantially regulate various signaling pathways, and the downregulation of tumor-suppressive miRs and the upregulation of oncomiRs have been associated with tumor development (14). Recent studies have shown that ectopic expression of specific PD-L1-targeting miRs in cancerous cells, like colorectal cancer and triple-negative breast cancer cells, can suppress tumor-intrinsic PD-L1 expression, stimulate anti-tumoral immune responses, substantially inhibit oncogenic signaling pathways, and decrease tumor development (12, 15). Accumulating studies have identified PD-L1-inhibiting miRs that some of them can suppress NSCLC development (6, 16–18). Xie et al. have shown that the ectopic expression of miR-140, as a PD-L1-inhibiting miR, is associated with decreased expression of cyclin E and suppressed cell proliferation in NSCLC cells (19). Chen et al. have reported that miR-526b-3p increased expression downregulates the expression of c-Myc and MDR1 and enhances cisplatin-cytotoxicity in NSCLC cells (16). Song et al. have shown that increased expression of hsa-miR-138-5p decreases tumor proliferation and arrests the cell cycle of NSCLC cells *via* downregulating CCND3, Ki67, CDC20, and MCM2 (20). However, there is no study to comprehensively collate and investigate their role in NSCLC development.

In the current study, we aimed to thoroughly and systematically search the literature to identify the current knowledge of the significance of PD-L1-inhibiting miR ectopic expression in NSCLC development. We also leveraged the cancer genome atlas (TCGA) data to study their clinical significance in the affected patients. Besides searching the literature, we leveraged *in silico* data to predict the circular RNA (circRNA)/miR/PD-L1 axes in NSCLC, which might translate into introducing novel therapeutic approaches. Finally, we applied single-cell RNA sequencing to study the potential results of the ectopic expression of PD-L1-inhibiting miRs on the immune cells residing in the NSCLC microenvironment.

## Materials and methods

### Scoping review of literature

#### Scoping review protocol

We applied Arksey and O’Malley’s framework (21), which was improved by Levac et al. (22). Also, the current scoping review is consistent with the preferred reporting items for systematic reviews and meta-analyses extension for scoping reviews (PRISMA-ScR) guidelines (23). The five steps of this scoping review consist of developing the research question, finding the relevant papers, selecting papers, data charting, and summarizing and reporting the obtained results.

#### The research question

Concerning the oncogenic role of tumor-intrinsic PD-L1 in developing colorectal cancer and triple-negative breast cancer (12, 15), the current scoping review aimed to study the current knowledge on the therapeutic potentiality of tumor-intrinsic PD-L1-inhibiting miRs to suppress NSCLC development.

#### Finding the published records

The Embase, Scopus, Web of Science, PubMed, Ovid, EBSCO, ProQuest, Cochrane Library, African Index Medicus, and Pascal-Francis were systematically searched to identify the published papers before 4 April 2021 without any restriction on language, country, and time. The following keywords were used to identify the relevant papers: (“programmed death-ligand 1” OR “PD-L1” OR “P.D. L1” OR “PDL1” OR “B7-H1” OR “B7 H1” OR “B7H1” OR “CD274” OR “cluster of differentiation 274” OR “CD 274” OR “cluster of differentiation274” OR “B7 homolog 1” OR “PDCD1 ligand 1” OR “PDCD1LG1” OR “PDCD1L1” OR “HPD-L1” OR “B7-H1 antigen” OR “programmed death 1 ligand 1”) and (“microRNA-” OR “microRNA” OR “micro RNA-” OR “micro RNA” OR “miRNA-” OR “miRNA” OR “miR-” OR “miR” OR “miRNA-” OR “miRNA” OR “MicroRNAs”) and (“NSCLC” OR “lung tumor” OR “lung malignancy” OR “lung” OR “non-small-cell lung carcinoma” OR “non-small-cell lung cancer” OR “non-small-cell lung carcinoma” OR “non-small cell lung carcinoma” OR “non-small cell lung carcinoma” OR “non-small-cell lung cancer” OR “non-small cell lung cancer” OR “squamous cell carcinoma of lung” OR “lung squamous cell carcinoma” OR “SCC” OR “large cell carcinoma” OR “carcinoma, non-small-cell lung” OR “lung neoplasms” OR “Adenocarcinoma of Lung” OR “Carcinoma, Large Cell” OR “Carcinoma, Squamous Cell” OR “lung cancer” OR “lung tumor” OR “non-small cell lung cancer” OR “adenocarcinoma” OR “lung adenocarcinoma” OR “squamous cell carcinoma”). The medical subject headings (MeSh) and Emtree terms were used in our systematic search to increase the sensitivity of the systematic search.

#### Study selection

Two independent co-authors (MS and FG) thoroughly reviewed the obtained records in two distinct phases. In the first phase, those co-authors independently screened the title and abstract of the retrieved records. In the second phase, they independently reviewed the full text of the remaining papers. The senior author, i.e., BB, was consulted to resolve any disagreements.

The included papers in the current scoping review must meet the following criteria, i.e., the included studies must be original articles published in English, and those papers must study the effect of miR ectopic expression on the expression of tumor-intrinsic PD-L1 in human NSCLC cells. The following records were excluded from the current scoping review: (I) duplicated records, (II) review articles, (III) meeting abstracts, (IV) editorial papers, (V) non-English papers, (VI) viewpoints, (VII) case reports, (VIII) book chapters, (IX) notes, (X) erratum, and (XI) the papers that did not meet the aforementioned inclusion criteria.

#### Data charting

We extracted the following results from the included papers: the first author, publication year, studied miR(s), studied cell line, the effect of studied miR(s) on tumor development, and the proposed cross-talk with other coding and non-coding RNAs.

#### Summarizing and reporting the obtained results

Besides summarizing the findings of the included studies, the current scoping review discusses the findings regarding the effect of these PD-L1-inhibiting miRs on NSCLC development that have not been presented in the included studies. Also, we included *in silico* investigations to present more insights regarding the significance of the PD-1/PD-L1 axis based on single-cell RNA sequencing.

### *In silico* investigation

#### The significance of identified programmed death-ligand 1-inhibiting miRs on biological pathways

We used the miRPathDB v2.0 to access the WikiPathways dataset and analyze them (24). We adjusted our analyses based on a minimum of four significant miRs per pathway and strong experimental evidence.

#### Identifying the circRNA-miR-PD-L1 axes in non-small cell lung cancer

We also studied the circRNA/miR/PD-L1 axis in NSCLC cancer patients. We accessed the GSE158695 and GSE63805 datasets *via* the Gene Expression Omnibus (GEO) database^1^ to identify the significantly upregulated circRNAs and downregulated miRs in NSCLCs, respectively. Then, we used the data of microT-CDS to predict the potential miRs that can target PD-L1. The common miRs between these are selected for further analyses. After that, we accessed the circMine^2^ to identify the targets of the identified significantly upregulated circRNA in NSCLC tissues. The | log_2_FC| ≥ 1 and adjusted *P*-value < 0.05 were the cut-offs for considering significantly differentially expressed miRs and circRNAs.

#### The prognostic significance of identified programmed death-ligand 1-inhibiting miRs in patients with lung adenocarcinoma

We accessed the TCGA-LUAD and TCGA-LUSC data *via* the UCSC Cancer Browser^3^. In our survival analyses, we only included primary tumor tissues and considered the upper quartile as “high” expression and the lower quartile as “low” expression.

#### Single-cell RNA sequencing

After highlighting the significance of PD-L1-inhibiting miRs on NSCLC development and biological pathways, we aimed to investigate which cells might be potentially liberated from the immunosuppressive PD-L1/PD-1 axis in the NSCLC microenvironment. For this purpose, we accessed to GSE144945 dataset *via* Gene Expression Omnibus (GEO) database (see text footnote 1). This dataset applied single-cell RNA sequencing on 10 tissues for NSCLC patients using GPL16791 Illumina HiSeq 2500 (Homo sapiens) (25). After downloading the dataset, we used Seurat (version 4.1.0) R package to analyze the raw data (24). Cells with mitochondrial gene expression of < 10% and expressed genes of above 500 were included in our analyses. We scaled them and ran the principal component analysis after normalizing data, identifying variable genes, and integrating data from multiple samples. Then, the uniform manifold approximation and projection (UMAP) clustering algorithms with a resolution of 0.11 were used to cluster the cells into a two-dimensional figure. Afterward, the PanglaoDB dataset and the information demonstrated in the original study were used to annotate the identified clusters according to the pertained gene markers (25, 26). The applied gene markers are demonstrated in Table 1.

**TABLE 1:** Markers used for annotating the cell clusters.

Cell cluster	Gene markers
CD8^+^ T-cells	CD3D, CD3E, CD3G, CD8A, and CD8B
CD4^+^ Treg	CD3D, CD4, IL2RA, and FOXP3
Unknown	HSPA1B, HSPA1A, MTRNR2L12, HSPH1, and FOSB
NKT cells	FCGR3A, KLRB1, KLRF1, and KLRD1
B-cells	MS4A1, CD19, CD79A, and CD79B
NK cells	KLRB1, KLRC1, KLRD1, and KLRF1
DCs	CLEC4C and IL3RA
ILC2	IL1RL1 and KIT
T-cells	IL7R, CCR7, LTB, KLRB1, GPR183, SPOCK2, and ANXA1

## Results

### Included studies

**FIGURE 1:**
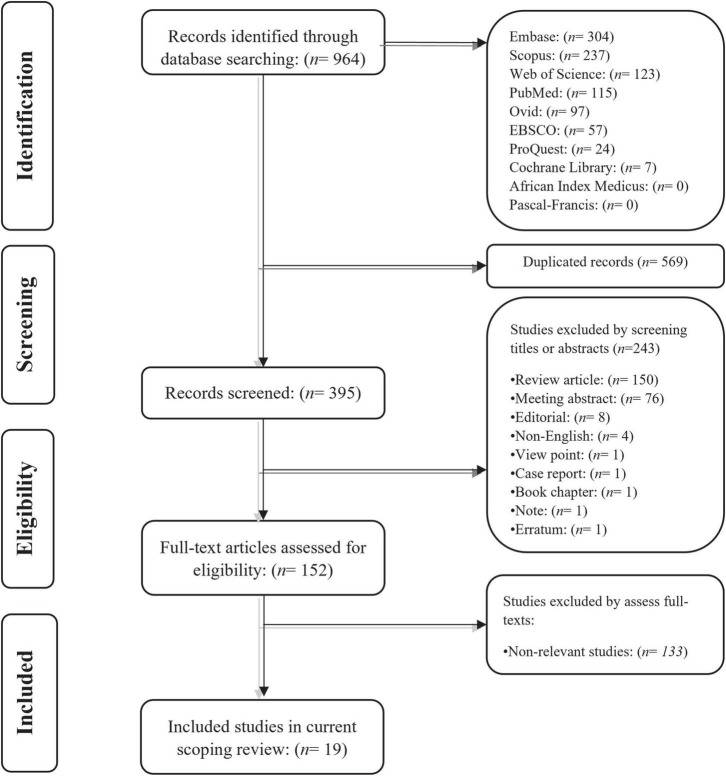
The study identification flowchart.

Our systematic search on Embase, Scopus, Web of Science, PubMed, Ovid, EBSCO, ProQuest, Cochrane library, African Index Medicus, and Pascal-Francis databases retrieved 964 potentially relevant records for the current scoping review. Following removing the duplicated records, the title and abstract of the remaining 395 papers were screened independently by two co-authors. After removing the records that were not suitable for the scoping review, those co-authors independently reviewed the full text of the remaining papers for consideration to be included in the study. Finally, 19 original studies were included in this scoping review. The literature identification flowchart is shown in Figure 1.

### The characteristics of the included studies

The 19 included studies were published between 2014 and 2021. A549 is the most studied NSCLC cell line in the included studies (Table 2). Our results have indicated that the increased expression of hsa-miR-142-5p, hsa-miR-155-5p, hsa-miR-194-5p, hsa-miR-326, hsa-miR-526b-3p, hsa-miR-138-5p, hsa-miR-377-3p, hsa-miR-200b-5p, hsa-miR-34a-5p, hsa-let-7c-5p, hsa-miR-200a-3p, hsa-miR-200c-3p, hsa-miR-140, hsa-miR-34b, hsa-miR-34c-5p, hsa-miR-197-3p, and hsa-miR-200b-3p inhibit tumor-intrinsic PD-L1 in NSCLC cells (Table 2).

**TABLE 2:** The characteristics of the included studies.

No.	References	Identified miR	Cell line	miR effect on NSCLC development
1	Zhu et al. (6)	hsa-miR-142-5p	A549 and HCC827 cells	FGD5-AS1 inhibits hsa-miR-142-5p expression, and hsa-miR-142-5p downregulates PD-L1 expression in NSCLC cells. PD-L1 knockdown and hsa-miR-142-5p ectopic expression decreased the migration and invasion of cisplatin-resistance NSCLC cells; however, the transfection of hsa-miR-142-5p inhibitor reversed this anti-tumoral effect.
2	Yang et al. (18)	hsa-miR-155-5p and hsa-miR-194-5p	A549 and H1395 cells	CircCHST15 inhibits the expression of hsa-miR-155-5p and hsa-miR-194-5p; hsa-miR-155-5p and hsa-miR-194-5p decrease PD-L1 expression in NSCLC cells. Although hsa-miR-155-5p increases the proliferation and clonogenicity of NSCLC cells, hsa-miR-194-5p decreases their proliferation and clonogenicity.
3	Shao et al. (17)	hsa-miR-326	A549 and H1734 cells	hsa-miR-326 expression is reduced in human NSCLC cell lines, i.e., A549 and H1734, and H1975, compared to normal human epithelial lung cells. hsa-miR-326 expression level is substantially decreased in NSCLC tissues, and there is a negative association between PD-L1 and hsa-miR-326 expression in primary and metastatic NSCLC tissues. hsa-miR-326 ectopic expression downregulates the expression of PD-L1 and B7-H3 in NSCLC cells. The expression of TNF-α, IFN-γ, and IL-2 are increased in the supernatant of hsa-miR-326 overexpressed cells. However, IL-1β, IL-10, and TGF-β expression are decreased. Also, the TNF-α^+^IFN-γ^+^CD8^+^ T-cells population is increased after hsa-miR-326 ectopic expression. Although hsa-miR-326 ectopic expression does not alter the proliferation of tumoral cells, it decreases the migration of tumoral cells. However, hsa-miR-326 expression decreases tumor growth, metastasis, and PD-L1 expression in animal models. Besides, ectopic expression of hsa-miR-326 increases the infiltration of CD8 cells and TNF-α^+^IFN-γ^+^CD8^+^ T-cells population in tumor tissues in animal models.
4	Chen et al. (16)	hsa-miR-526b-3p	A549 and PC-9	miR-526b-3p is substantially decreased in cisplatin-resistant lung cancer tissues compared to cisplatin-sensitive tissues. Also, the expression of miR-526b-3p is substantially decreased in NSCLC cell lines, i.e., H1975, A549, and PC-9, compared to normal human epithelial lung cells. miR-526b-3p increases cisplatin chemosensitivity and decreases the migration and proliferation of NSCLC cells. Also, miR-526b-3p ectopic expression increases the population of CD8^+^ T-cells. Besides, miR-526b-3p ectopic expression decreases the expression of PD-L1, MDR1, c-Myc, and STAT3.
5	Wang et al. (42)	hsa-miR-34a-5p	95D	circRNA-002178, which is upregulated in tumoral cells compared to normal cells, sponges hsa-miR-34a-5p expression and liberates the PD-L1 mRNA from the inhibitory effect of hsa-miR-34a-5p. The exosomal plasmatic level of circRNA-002178 can be a useful diagnostic marker for LUAD patients (AUC = 0.9956, and *P*-value < 0.001). The exosomal circRNA-002178 transferred from tumoral cells is significantly enriched in the tumor-infiltrating CD8^+^ T-cells of affected patients and sponges hsa-miR-28-5p expression to increase PD-1 expression in CD8^+^ T-cells.
6	Song et al. (20)	hsa-miR-138-5p	A549	hsa-miR-138-5p inhibits NSCLC development, increases tumor-infiltrating mature DCs, CD4^+^ T-cells, and CD8^+^ T-cells, and decreases tumor cell proliferation and tumor-infiltrating regulatory DCs. hsa-miR-138-5p downregulates the protein expression of tumor-intrinsic PD-L1 expression and PD-1 and PD-L1 expression in tumor-infiltrating DCs. However, hsa-miR-138-5p does not affect the mRNA expression of tumor-intrinsic PD-L1 in A549 and 3LL cells. hsa-miR-138-5p decreases PD-1 expression in DCs and T-cells, and miR-138-5p increases the ability of DC to induce cytotoxicity of CD8^+^ T-cells and promote the proliferation of CD4^+^ and CD8^+^ T-cells.
7	Li et al. (46)	hsa-miR-377-3p	A549	circRNA_0000284 targets hsa-miR-377-3p to increase PD-L1 expression. circRNA_0000284 expression is upregulated in NSCLC tissues compared to non-tumoral adjacent tissues, in higher stage (stage III and stage IV) tumor tissues than lower stage (stage I and stage II) tumor tissues, and in tissues with lymph node metastasis than tissues without lymph node metastasis. The increased expression of circRNA_0000284 has been associated with the decreased overall survival rate of affected patients. hsa-miR-377-3p targets tumor-intrinsic mRNA and protein PD-L1 expression in A549 and H82 cells. The pro-tumoral effect of circRNA_0000284 on cell proliferation, colony formation, migration, and invasion is mediated *via* the hsa-miR-377-3p/PD-L1 axis.
8	Katakura et al. (57)	hsa-miR-200b-5p	H460	hsa-miR-200b-5p inhibits tumor-intrinsic PD-L1 expression.
9	Kang et a. (44)	hsa-miR-34a-5p	A549 and H292	hsa-miR-34a-5p inhibitor increases tumor-intrinsic PD-L1 expression.
10	Huang et al. (53)	hsa-miR-155-5p	A549 and H1650	hsa-miR-155-5p inhibits tumor-intrinsic PD-L1 expression. Also, there is a negative correlation between hsa-miR-155-5p and PD-L1 expression in LUAD tissues.
11	Hong et al. (41)	hsa-let-7c-5p	A549 and H1299	circ-CPA4 targets hsa-let-7c-5p and liberate PD-L1 expression in NSCLC. In NSCLC tissues and cells, the expression of circ-CPA4 and PD-L1 is upregulated, and hsa-let-7c-5p expression is downregulated. Anti-PD-L1 antibodies interfere with the protective effect of the NSCLC cell-originated exosomes in cisplatin-induced cell death of NSCLC cancer cells. Tumor-intrinsic PD-L1 silencing or PD-L1/PD-1 blockade increased the proliferation and viability of CD8^+^ T-cells in a co-culture system. Besides, PD-L1 knockdown or blockade upregulated the expression of IFN-γ and IL-4 and downregulated IL-10 expressions in CD8^+^ T-cells.
12	Chen et al. (52)	hsa-miR-155-5p	A549	hsa-miR-155-5p inhibits tumor-intrinsic PD-L1 expression in NSCLC cells and decreases tumor cell proliferation. hsa-miR-155-5p increases the infiltration of IFN-γþ lymphocytes, CD4þ T lymphocytes, and CD8þ T lymphocytes. Also, hsa-miR-155-5p increases the expression of TNF-a and IFN-g and downregulates IL-10 expression.
13	Wei et al. (49)	hsa-miR-200a-3p	A549 and CAL-12T	MALAT1 sponges hsa-miR-200a-3p to liberate PD-L1 protein expression in NSCLC cells. MALAT1 overexpression increases cell proliferation, clonogenicity, migration, and invasion and decreases apoptosis in NSCLC cells; however, hsa-miR-200a-3p overexpression partially reverses the pro-tumoral effect of MALAT1 ectopic expression.
14	Dai Phung et al. (54)	hsa-miR-200c-3p	NCI-H1299	hsa-miR-200c-3p downregulates PD-L1 mRNA and protein expression in NSCLC cells. hsa-miR-200c-3p potentiates doxorubicin-induced cytotoxicity in NSCLC cells and substantially increases the cytotoxic T-cell-mediated killing against NSCLC cells and IFN-γ production.
15	Xie et al. (19)	hsa-miR-140	A549 and NCI-H1650	hsa-miR-140 downregulates protein and mRNA expression of PD-L1 in NSCLC cells. PD-L1-silencing or hsa-miR-140 ectopic expression downregulates cyclin-E protein and mRNA expression levels in NSCLC cells. Also, hsa-miR-140 overexpression arrests the cell cycle and decreases the proliferation of NSCLC cells.
16	Wan et al. (59)	hsa-miR-142-5p	A549	Increased expression of hsa-miR-142-5p inhibits PD-L1 expression in NSCLC cells. The overexpression of hsa-miR-142-5p increases tumor growth and decreases the apoptosis rate in NSCLCs, and anti-hsa-miR-142-5p ectopic expression has reversed these pro-tumoral effects.
17	Cortez et al. (43)	hsa-miR-34a-5p, hsa-miR-34b, and hsa-miR-34c-5p	A549, H460, and H1299	Increased expression of hsa-miR-34a-5p, hsa-miR-34b, and hsa-miR-34c-5p can inhibit PD-L1 protein expression in NSCLC cells.
18	Fujita et al. (55)	hsa-miR-197-3p	A549, and PC14	hsa-miR-197-3p is substantially downregulated in NSCLC cells compared to normal bronchial cells, and its ectopic expression enhances the chemosensitivity of NSCLC cells *in vitro*. hsa-miR-197-3p can suppress PD-L1 expression in NSCLC cells. The inhibition of hsa-miR-197-3p increases tumor growth and metastasis and worsens the survival of affected animal models.
19	Chen et al. (50)	hsa-miR-200b-3p and hsa-miR-200a-3p	H1299	hsa-miR-200b-3p and hsa-miR-200a-3p can downregulate PD-L1 expression in NSCLC cells.

### The significance of the identified programmed death-ligand 1-inhibiting miRs on biological pathways

**FIGURE 2:**
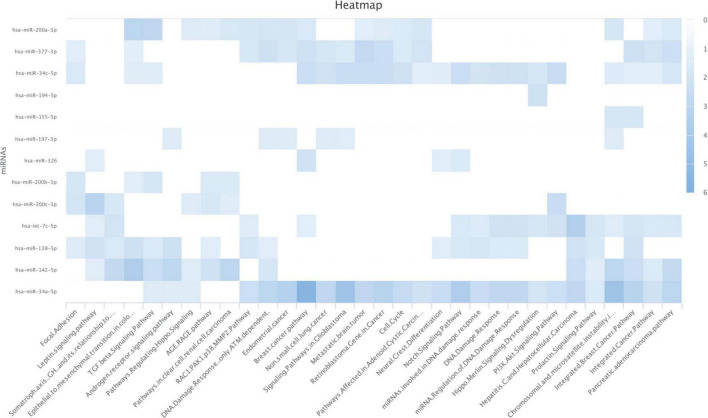
Enrichment analyses on the identified programmed death-ligand 1 (PD-L1)-inhibiting miRs based on the WikiPathways. The darker color shows a more significant one.

We used the WikiPathways dataset to analyze the significance of the identified PD-L1-inhibiting miRs on biological pathways. Our results have indicated that hsa-miR-34a-5p, hsa-miR-377-3p, hsa-miR-200a-3p, and hsa-miR-34c-5p are significantly enriched for regulating the cell cycle (Figure 2). Also, our results have indicated that hsa-miR-34a-5p, hsa-let-7c-5p, hsa-miR-200c-3p, and hsa-miR-34c-5p are significantly enriched for regulating the PI3K/Akt signaling pathway (Figure 2).

### The prognostic values of the identified programmed death-ligand 1-inhibiting miRs in patients with lung adenocarcinoma and lung squamous cell carcinoma

**FIGURE 3:**
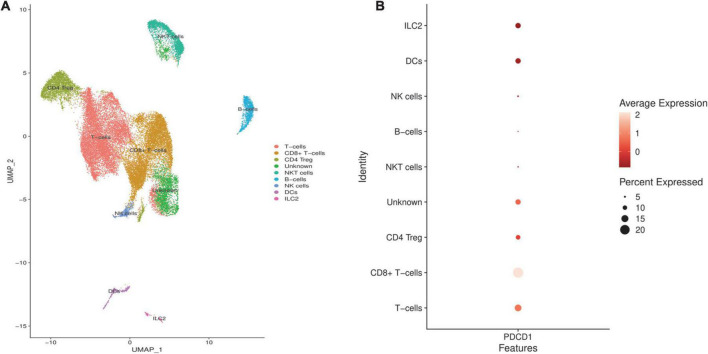
The prognostic values of hsa-miR-200a-3p and hsa-let-7c-5p, as programmed death-ligand 1 (PD-L1)-inhibiting miRs, based on TCGA-LUAD and TCGA-LUSC databases. **(A)** High expression of hsa-miR-200a-3p is associated with enhanced disease-specific survival of LUAD patients. **(B)** High expression of hsa-let-7c-5p is associated with improved disease-specific survival of patients with lung squamous cell carcinoma (LUSC).

Our results have indicated that high expression of hsa-miR-200a-3p, as a PD-L1-inhibiting miR, is significantly associated with improved disease-specific survival of patients with LUAD (*P*-value = 0.01844) (Figure 3A). Also, high expression of hsa-let-7c-5p, as a PD-L1-inhibiting miR, is significantly associated with enhanced disease-specific survival of patients with LUSC (*P*-value = 0.001504) (Figure 3B).

### The circRNA/miR/PD-L1 axes in non-small cell lung cancer

**FIGURE 4:**
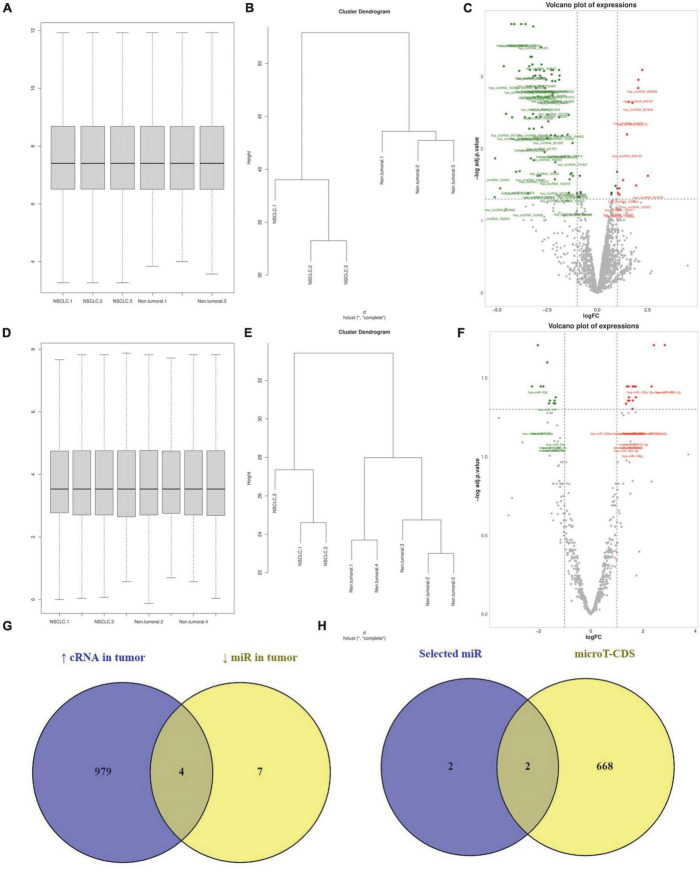
has_circ_0002476/hsa-miR-216a-5p/PD-L1 and hsa_circ_0063526/hsa-miR-1299/PD-L1 might increase programmed death-ligand 1 (PD-L1) expression in non-small cell lung cancer (NSCLC). **(A–C)** Normalizing, clustering, and identifying differentially expressed circRNAs from the GSE158695 dataset. **(D–F)** Normalizing, clustering, and identifying differentially expressed circRNAs from the GSE63805 dataset. **(G)** Identifying the commonality between the miR targets of upregulated circRNAs in NSCLC tissues between the downregulated miRs in NSCLC tissues. **(H)** Identifying which of those miRs can target PD-L1 based on the microT-CDS database.

We analyzed the GSE158695 dataset to identify significantly upregulated circRNAs in NSCLC tissues compared to normal lung tissues. For this aim, we normalized the expression values of 3 NSCLC and 3 non-tumoral tissues (Figure 4A). Our results have shown that the expression profile of the included tissues is consistent with two distinct patterns (Figure 4B). We have identified 12 upregulated circRNAs in NSCLC tissues (Figure 4C).

In the same manner, we analyzed the GSE63805 dataset to identify significantly downregulated miRs in NSCLC tissues. After data normalization and clustering, our results have demonstrated 11 significantly downregulated miRs in NSCLC tissues (Figures 4D–F, respectively). We have demonstrated that 4 miR-targets of those 12 upregulated circRNAs are among the 11 significantly downregulated miRs; these miRs are hsa-miR-216a-5p, hsa-miR-144-3p, hsa-miR-1299, and hsa-miR-1909-3p (Figure 4G). Based on microT-CDS, hsa-miR-216a-5p and hsa-miR-1299 might directly target PD-L1 expression (Figure 4H). Therefore, our *in silico* results suggested that has_circ_0002476/hsa-miR-216a-5p/PD-L1 and hsa_circ_0063526/hsa-miR-1299/PD-L1 might be two axes in NSCLC that might increase PD-L1 expression in NSCLC tissues.

### The expression pattern of PD-1 in non-small cell lung cancer microenvironment

**FIGURE 5:**
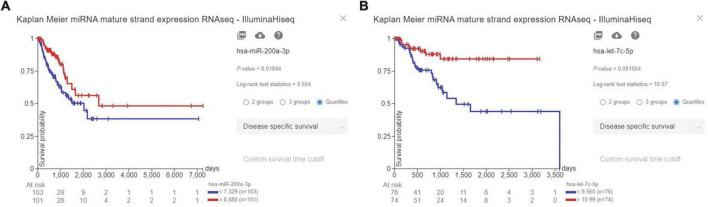
Identifying programmed death-ligand 1 (PD-L1) expression pattern in the included non-small cell lung cancers (NSCLCs) microenvironment. **(A)** The clustering and cell annotation displayed nine cell clusters in the included tumor microenvironment of NSCLCs. **(B)** PD-1 is considerably expressed in the T-cells, CD8^+^ T-cells, CD4 Treg, DCs, and ILC2 in the included NSCLCs microenvironment.

We analyzed the GSE144945 dataset to identify the expression pattern of PD-1 in the NSCLC microenvironment and highlight which cell populations might be liberated from the tumor-intrinsic PD-L1 inhibition in the NSCLC microenvironment. After excluding low-quality cells, we included 32,116 cells from 10 NSCLC tissues. Based on the abovementioned markers, we identified 9 cell populations residing in the included NSCLC microenvironment (Figure 5A). Our results have indicated that PD-1 might be considerably expressed in the T-cells, CD8^+^ T-cells, CD4 regulatory T-cells (Tregs), DCs, and ILC2 in the included tumor microenvironment of NSCLCs (Figure 5B).

## Discussion

In 2022, lung cancer will be the second most commonly diagnosed and the most common cause of cancer-related death in men and women in the United States (1). NSCLC is responsible for the majority of lung cancer cases, and most patients are diagnosed when the disease is advanced (27). Although there have been notable advances in treating NSCLCs, disease progression and resistance development have been considerable burdens for affected patients (28). In this regard, a better understanding of NSCLC biology and its microenvironment can pave the way for developing novel treatments for affected patients.

As small non-coding RNAs, miRs have pivotal roles in the post-transcriptional regulation of genes. It is well-established that miRs can bind to the 3’ untranslated region (UTR) of target mRNAs and facilitate translation inhibition; therefore, they are involved in regulating various biological processes (29). Growing evidence has highlighted that specific miRs can pave the way for tumor development, and other miRs can inhibit tumor development and metastasis (12, 15). Tumor-suppressive miRs target the expression of oncogenes, while oncomiRs target the expression of tumor-suppressive genes. In this regard, the ectopic expression of tumor-suppressive miRs and suppressing the expression of oncomiRs can inhibit tumor development and migration (30). Since miRs can regulate a vast number of gene expressions, both directly and indirectly, they carry valuable therapeutic potential for cancer treatment (31).

Recently, circRNAs have emerged as a new class of non-coding of RNAs that considerably regulate gene expression (32). It has been reported that circRNAs are involved with miR sponges, protein/peptide translators, RNA-binding protein sponges, and regulating gene splicing and transcription (31). One of the most studied circRNA-mediated interactions is the cross-talk between circRNA and miRs; circRNAs have multiple sites for binding miRs that can “sponge” them and therefore regulate the expression of various genes (33). Thus, identifying the circRNA/miR/mRNA axes that have significant roles in cancer development/inhibition can be a milestone in cancer treatment.

Immunotherapy is one of the novel and promising treatments for a subset of cancer patients. Stimulating the host’s anti-tumoral immune responses is the cornerstone of immunotherapeutic approaches (3). Effective rejection of tumors can also eradicate cancerous cells and prevent tumor relapse; however, the immunosuppressive tumor microenvironment has been implicated in tumor development and shielding tumoral cells from anti-tumoral immune responses (34). Inhibitory immune checkpoint axes considerably attenuate anti-tumoral immune responses and contribute to tumor growth (3). The PD-1/PD-L1 axis is one of these inhibitory axes that can maintain this pathological immune tolerance against neoplastic cells (3). In this regard, multiple clinical studies have demonstrated the beneficial effect of administrating monoclonal antibodies that inhibit the PD-1/PD-L1 axes in NSCLC patients. Following the promising results, the FDA has approved pembrolizumab (anti-PD-1), nivolumab (anti-PD-1), atezolizumab (anti-PD-L1), and durvalumab (anti-PD-L1) for patients with NSCLC. Thus, targeting this inhibitory axis is pivotal in treating NSCLC (7).

Besides inhibiting anti-tumoral immune responses, recent findings have indicated that tumor-intrinsic inhibitory immune checkpoint molecules have oncogenic effects in various cancers, like triple-negative breast cancer, NSCLC, and melanoma (35–38). In TNBC cells, PD-L1 knockdown has substantially decreased cell viability, stemness, cell cycle progression, and migration. Also, tumor-intrinsic PD-L1 silencing has stimulated apoptosis of TNBC cells and decreased the CD25^+^ forkhead box P3 (Foxp3)^+^ Tregs (36). Qiu et al. have reported that PD-L1 overexpression has substantially increased cell proliferation and migration in glioblastoma cells *via* the mitogen-activated extracellular signal-regulated kinase (MEK)/extracellular signal-related kinase (Erk) pathway both *in vitro* and *in vivo* (39). Wei et al. have reported that increased expression of PD-L1 increases the self-renewal of colorectal cancer cells and PD-L1 knockdown decreases colorectal cancer stemness (40). Yu et al. have demonstrated that PD-L1 knockdown has substantially decreased cell viability and reduced tumor clonogenicity and migration in NSCLC cells (35). Hong et al. have shown that tumor-intrinsic PD-L1 silencing or PD-L1/PD-1 blockade can increase the proliferation and viability of CD8^+^ T-cells, upregulate the expression of IFN-γ and IL-4, and downregulate IL-10 expression in a co-culture system with NSCLC cells (41). Therefore, inhibiting tumor-intrinsic PD-L1 can stimulate anti-tumoral immune responses and suppress oncogenic signaling pathways, leading to suppressed tumor growth.

Considering the inhibitory effect of tumor-intrinsic PD-L1 on anti-tumoral immune responses, the established oncogenic effect of tumor-intrinsic PD-L1 on tumor development, and the multi-target and considerable effect of miRs on mRNAs expression, identifying PD-L1-inhibiting miRs and studying the overall effect of their ectopic expression on tumoral cells can pave the way for the development of novel therapeutic approaches. It has been reported that the ectopic expression of hsa-miR-497-5p, hsa-miR-424-5p, hsa-miR-34a-5p, hsa-miR-200c-3p, hsa-miR-138-5p, hsa-miR-383-5p, hsa-miR-195-5p, hsa-miR-570-3p, and hsa-miR-3609 can considerably inhibit tumor proliferation, migration, stemness, tumor-intrinsic PD-L1 expression in TNBC cells. Besides contributing to the transformation of the tumor microenvironment from immunosuppressive to pro-inflammatory, their ectopic expression has been associated with enhanced chemosensitivity in TNBC cells (15). Consistent with this, it has been shown that ectopic expression of hsa-miR-140-3p, hsa-miR-382-3p, hsa-miR-148a-3p, hsa-miR-93-5p, hsa-miR-200a-3p, hsa-miR-200c-3p, hsa-miR-138–5p, and hsa-miR-15b-5p can suppress PD-L1 expression and inhibit tumor proliferation and migration in colorectal cancer cells; these PD-L1-inhibiting miRs substantially transform the tumor microenvironment from immunosuppressive to pro-inflammatory and improve the chemosensitivity of colorectal cancer cells regardless of the microsatellite state (12). In the current study, we aimed to identify the effect of PD-L1-inhibiting miRs on NSCLC development and investigate the circRNA/miR/PD-L1 axes based on the literature and *in silico* data.

Zhu et al. have identified the FGD5-AS1/hsa-miR-142-5p/PD-L1 axis in NSCLC cells. They have reported that FGD5-AS1 inhibits hsa-miR-142-5p expression, and hsa-miR-142-5p ectopic expression downregulates PD-L1 expression and decreases the migration and invasion of cisplatin-resistant NSCLC cells (6). Yang et al. have identified the circCHST15/hsa-miR-194-5p/PD-L1 axis in NSCLC cells; they have shown that circCHST15 inhibits the expression of hsa-miR-194-5p and hsa-miR-194-5p ectopic expression decreases the proliferation and clonogenicity of NSCLC cells (18). Wang et al. have identified the circRNA-002178/hsa-miR-34a-5p/PD-L1 axis in NSCLC cells; circRNA-002178 inhibits the expression of hsa-miR-34a-5p, leading to the liberation of PD-L1 expression. Of interest, it has been reported that the exosomal plasmatic level of circRNA-002178 has a significant diagnostic value in LUAD patients (AUC = 0.9956, and *P*-value < 0.001) (42). Consistent with this, increased expression of hsa-miR-34a-5p mimic and hsa-miR-34a-5p inhibitor have decreased and increased PD-L1 expression in NSCLC cells (43, 44). It has been reported that hsa-miR-34a-5p overexpression inhibits the proliferation, migration, and invasion of LUAD cells. Also, hsa-miR-34a-5p overexpression stimulates apoptosis of LUAD cells (45). We have shown that hsa-miR-34a-5p is significantly enriched for regulating the PI3K/Akt pathway and cell cycle. Li et al. have highlighted the circRNA_0000284/hsa-miR-377-3p/PD-L1 axis in NSCLC cells, and the protumoral effect of circRNA_0000284 on cell proliferation, clonogenicity, migration, and invasion of NSCLC cells is mediated *via* the hsa-miR-377-3p/PD-L1 axis. They have shown that the expression level of circRNA_0000284 is substantially upregulated in NSCLC tissues compared to non-tumoral tissues, in higher stage NSCLC tissues compared to lower stage NSCLC tissues, in tissues with lymph node metastasis compared with the tissues without lymph node metastasis. Of interest, circRNA_0000284 expression level has been associated with the inferior overall survival of affected patients (46). Besides, it has been shown that hsa-miR-377-3p decreases the cell viability, migration, and invasion of NSCLC cells (47). We have shown that hsa-miR-377-3p is significantly enriched for regulating the cell cycle. Hong et al. have shed light on the circ-CPA4/hsa-let-7c-5p/PD-L1 axis in NSCLC cells; they have shown that circ-CPA4 targets hsa-let-7c-5p and liberate PD-L1 expression in NSCLC (41). Also, it has been reported that hsa-let-7c-5p suppresses the cell viability of NSCLC cells (48). Our results have indicated that increased expression of hsa-let-7c-5p is associated with improved disease-specific survival of LUSC patients. Also, we have demonstrated that hsa-let-7c-5p is significantly enriched for regulating the PI3K/Akt pathway. Wei et al. have highlighted the MALAT1/hsa-miR-200a-3p/PD-L1 axis in NSCLC cells; they have shown that MALAT1 sponges hsa-miR-200a-3p to liberate PD-L1 protein expression in NSCLC cells and MALAT1 overexpression increases clonogenicity, cell migration, proliferation, and invasion and decrease apoptosis in NSCLC cells. However, hsa-miR-200a-3p ectopic expression partially reverses the pro-tumoral effect of MALAT1 in NSCLC cells (49). Chen et al. have reported that hsa-miR-200a-3p suppresses tumor-intrinsic PD-L1 expression in NSCLC cells (50). Tan et al. have reported that ectopic expression of hsa-miR-200a-3p decreases the proliferation, invasion, and migration of NSCLC cells (51). Our results have shown that increased expression of hsa-miR-200a-3p is associated with enhanced disease-specific survival of LUAD patients. Besides, we have shown that hsa-miR-200a-3p is significantly enriched for regulating the cell cycle. Yang et al. have highlighted the CircCHST15/hsa-miR-155-5p/PD-L1 axis in NSCLC cells; circCHST15 suppresses hsa-miR-155-5p expression and liberates PD-L1 expression in NSCLC cells. Yang et al. have reported that hsa-miR-155-5p ectopic expression increases the proliferation and clonogenicity in H1395 and A549 cells (18). Consistent with this, Chen et al. have reported that hsa-miR-155-5p transfection decreases PD-L1 expression; however, Chen et al. have reported that hsa-miR-155-5p transfection suppresses the proliferation of A549 cells. They have shown that hsa-miR-155-5p increases the infiltration of IFN-γþ lymphocytes, CD4þ T lymphocytes, and CD8þ T lymphocytes, upregulates the expression of TNF-α and IFN-g, and downregulates IL-10 expression (52). In line with these, Huang et al. have demonstrated that hsa-miR-155-5p targets mRNA and protein PD-L1 expression in A549 and H1650 cells. Also, they have shown a negative correlation between hsa-miR-155-5p expression and PD-L1 expression in LUAD tissues (53).

It has been reported that hsa-miR-326 ectopic expression downregulates PD-L1 and B7-H3 expression in NSCLC cells. Besides, there have been upregulation in TNF-α, IFN-γ, and IL-2 expression and downregulation in IL-1β, IL-10, and TGF-β expression in the supernatant of hsa-miR-326 overexpressed cells. The overexpression of hsa-miR-326 has substantially decreased tumor growth, metastasis, and PD-L1 expression in animal models and increased the infiltration of CD8^+^ cells and TNF-α^+^ IFN-γ^+^ CD8^+^ T-cells population in tumor tissues in animal models of NSCLC (17). Chen et al. have demonstrated that miR-526b-3p can enhance cisplatin chemosensitivity, decrease the migration and proliferation of NSCLCs, and increase the population of CD8^+^ T-cells (16). Song et al. have reported that hsa-miR-138-5p inhibits NSCLC proliferation, decreases PD-L1 protein expression, and increases tumor-infiltrating mature DCs, CD4^+^ T cells, and CD8^+^ T-cells. Also, hsa-miR-138-5p downregulates PD-1 and PD-L1 expression in tumor-infiltrating DCs and increases the ability of DCs to induce cytotoxicity of CD8^+^ T-cells (20). It has been shown that hsa-miR-200c-3p downregulates PD-L1 mRNA and protein expression in NSCLC cells, and hsa-miR-200c-3p potentiates doxorubicin-induced cytotoxicity in NSCLC cells. Also, hsa-miR-200c-3p considerably enhances IFN-γ expression and the cytotoxic T-cell-mediated anti-tumoral effect against NSCLC cells (54). We have shown that hsa-miR-200c-3p is significantly enriched for regulating the PI3K/Akt pathway. Xie et al. have shown that miR-140 downregulates the protein and mRNA expression of PD-L1, arrests the cell cycle, and decreases the proliferation of NSCLC cells (19). Fujita et al. have demonstrated that the increased expression of hsa-miR-197-3p suppresses PD-L1 expression and enhances the chemosensitivity of NSCLC cells; hsa-miR-197-3p inhibition increases tumor growth and its migration and decreases the survival of affected animals (55). Cortez et al. have shown that hsa-miR-34c-5p and hsa-miR-34b suppress PD-L1 expression in NSCLC cells (43). It has been reported that hsa-miR-34c-5p increased expression decreases the migration and invasion of NSCLC cells (56). Our results have indicated that hsa-miR-34c-5p is significantly enriched for regulating the PI3K/Akt pathway and the cell cycle. It has been reported that hsa-miR-200b-3p and hsa-miR-200b-5p downregulate tumor-intrinsic PD-L1 expression in NSCLC cells (50, 57). The ectopic expression of hsa-miR-200b-3p has substantially decreased the migration and invasion of NSCLC cells (58). However, further studies are needed to clarify the overall effect of the hsa-miR-200b-5p ectopic expression on NSCLC cells. Despite the inhibitory role of hsa-miR-142-5p on tumor-intrinsic NSCLC cells, the overexpression of hsa-miR-142-5p increases tumor growth and decreases the apoptosis rate in NSCLC cells (59). In contrast, Jiang et al. have reported that hsa-miR-142-5p overexpression decreases cell proliferation, migration, and invasion and increases the apoptosis of NSCLC cells (60). Therefore, further studies are needed to evaluate the overall effect of the hsa-miR-142-5p ectopic expression on NSCLC development. Table 3 summarizes the tumor-suppressive PD-L1-inhibiting miRs in NSCLC.

**TABLE 3:** The tumor-suppressive PD-L1-inhibiting miRs in NSCLC.

miR	Ectopic expression effect on NSCLC development	References
hsa-miR-142-5p	Inhibiting the migration and invasion of cisplatin-resistance NSCLC cells.	(6)
hsa-miR-194-5p	Inhibiting the proliferation and clonogenicity of NSCLC cells	(18)
hsa-miR-326	Inhibiting the migration of NSCLC cells and stimulating inflammation	(17)
hsa-miR-526b-3p	Increasing cisplatin chemosensitivity and decreasing the migration and proliferation of NSCLC cells	(16)
hsa-miR-34a-5p	Inhibiting the proliferation, migration, and invasion and stimulating apoptosis	(45)
hsa-miR-138-5p	Inhibiting the proliferation of NSCLC cells and stimulating inflammation	(20)
hsa-miR-377-3p	Decreasing the cell viability, migration, and invasion of NSCLC cells	(47)
hsa-let-7c-5p	Decreasing the cell viability of NSCLC cells	(48)
hsa-miR-200a-3p	Decreasing the cell viability, migration, and invasion of NSCLC cells	(51)
hsa-miR-200c-3p	Potentiating doxorubicin-induced cytotoxicity in NSCLC cells and stimulating inflammation	(54)
hsa-miR-34c-5p	Decreasing the migration and invasion of NSCLC cells	(56)
hsa-miR-197-3p	Increasing the cisplatin and paclitaxel chemosensitivity of NSCLC	(55)
hsa-miR-200b-3p	Decreasing the migration and invasion of NSCLC cells	(58)

System biology approaches have provided valuable insights into the expression profile of various human conditions (61, 62). Following systematically reviewing the literature, we aimed to leverage the data obtained from system biology to predict the potential circRNA/PD-L1-targeting miRs in NSCLC tissues. Our *in silico* results have highlighted the hsa_circ_0002476/hsa-miR-216a-5p/PD-L1 and hsa_circ_0063526/hsa-miR-1299/PD-L1 in NSCLC. Based on our *in silico* analyses, hsa-miR-216a-5p and hsa-miR-1299 can be significantly downregulated in NSCLCs and can suppress PD-L1 expression. Also, hsa_circ_0002476 and hsa_circ_0063526 can be significantly upregulated in NSCLC tissues, and hsa_circ_0002476 and hsa_circ_0063526 can inhibit hsa-miR-216a-5p and hsa-miR-1299 expression. Therefore, hsa_circ_0002476 and hsa_circ_0063526 might sponge hsa-miR-216a-5p and hsa-miR-1299, respectively, liberating PD-L1 expression in NSCLC. Consistent with our *in silico* investigation, Pang et al. have shown that hsa-miR-216a-5p is substantially decreased in NSCLC cells and tissues compared to normal cells and tissues, respectively. Also, the ectopic expression of hsa-miR-216a-5p has substantially decreased the proliferation, invasion, and migration and stimulated apoptosis in NSCLC cells (63). Recently, Chai et al. have demonstrated that miR-1299 expression has substantially decreased in NSCLC tissues compared to normal tissues (64). In line with these, Cao et al. have also reported that miR-1299 expression is substantially decreased in NSCLC cells and tissues compared to non-tumoral cells and tissues, respectively. Also, miR-1299 ectopic expression suppresses proliferation, migration, invasion, and epithelial-mesenchymal transition processes and stimulates apoptosis in NSCLC cells by regulating the PI3K/Akt pathway (65).

Single-cell RNA sequencing can be considered a milestone in understanding various cells residing in the tumor microenvironment and their expression profile (66). This novel approach enables us to dissect each cell of tumor tissue, thoroughly examine its expression profile, categorize them into groups, and investigate the expression of a specific gene in these cell groups (67). Following identifying the significance of ectopic expression of PD-L1-inhibiting miRs in NSCLC development and discussing the underlying mechanisms through systematic literature review and tumor bulk analysis, we aimed to answer the question of which cells residing in the tumor microenvironment of NSCLC might be liberated following ectopic expression of PD-L1-inhibiting miRs in NSCLC cells. For this aim, we analyzed 32,116 cells from 10 NSCLC tissues to identify which cells express PD-1 in the tumor microenvironment of those NSCLC tissues. Based on our analyses, tumor-infiltrated T-cells, CD8^+^ T-cells, CD4 Treg, DCs, and ILC2 might considerably express PD-1; therefore, ectopic expression of hsa-miR-194-5p, hsa-miR-326, hsa-miR-526b-3p, hsa-miR-34a-5p, hsa-miR-34c-5p, hsa-miR-138-5p, hsa-miR-377-3p, hsa-let-7c-5p, hsa-miR-200a-3p, hsa-miR-200b-3p, hsa-miR-200c-3p, and hsa-miR-197-3p might stimulate CD8^+^ T-cells and DCs-mediated anti-tumoral immune responses *via* inhibiting tumor-intrinsic PD-L1 expression in NSCLC.

The current study has several limitations that should be noted. First, we only included papers published in English before the mentioned date. The second limitation stems from the intrinsic limitation of single-cell RNA sequencing approaches, i.e., the count of sequenced cells in our single-cell RNA sequencing was limited compared to the count of sequenced cells in tumor bulk analysis. Nevertheless, this study has strengths as well. First, we systematically searched ten databases, i.e., Embase, Scopus, Web of Science, PubMed, Ovid, EBSCO, ProQuest, Cochrane library, African Index Medicus, and Pascal-Francis, to retrieve available papers published before the mentioned data. Second, we included *in silico* tumor bulk and single-cell RNA sequencing to enrich this study. Third, we predicted circRNA/miR/PD-L1 axes based on bioinformatic analyses and discussed their significance based on a thorough literature review of NSCLC development.

## Conclusion

Overall, the ectopic expression of hsa-miR-194-5p, hsa-miR-326, hsa-miR-526b-3p, hsa-miR-34a-5p, hsa-miR-34c-5p, hsa-miR-138-5p, hsa-miR-377-3p, hsa-let-7c-5p, hsa-miR-200a-3p, hsa-miR-200b-3p, hsa-miR-200c-3p, and hsa-miR-197-3p, as PD-L1-inhibiting miR, can inhibit NSCLC development and stimulate anti-tumoral immune responses. Based on the single-cell RNA sequencing analyses, PD-1 might be considerably expressed on CD8^+^ T-cells and DCs; therefore, the ectopic expression of the abovementioned PD-L1-inhibiting miRs might stimulate the CD8^+^ T-cells and DCs-mediated anti-tumoral immune responses. Considering the multi-target nature of circRNA and miRs, identifying tumor-suppressive circRNA and miRs can provide a promising opportunity to overcome tumor growth, metastasis, and therapy resistance. Nevertheless, further studies are needed to identify, validate, and translate them into clinical practice.

## Data availability statement

Publicly available datasets were analyzed in this study. This data can be found here: geo databases, GSE158695, GSE63805, and GSE144945; and the TGCA database.

## Author contributions

MS: developing the research question, performing the *in silico* and single-cell RNA sequencing analyses, searching the databases, selecting the studies from the initial systematic search, and writing the manuscript. FG and HH: selecting the studies from the initial systematic search. NA and NH: extracting the results from selected studies. AD and NSh: searching the literature. OB: reviewing and editing the manuscript. NSi and BB: reviewing and editing the manuscript and supervising the study. All authors contributed to the article and approved the submitted version.

## Footnotes

1
https://www.ncbi.nlm.nih.gov/geo/

2
http://www.biomedical-web.com/circmine/home

3
https://xenabrowser.net/
